# Molecular Characterization of Transgene Integration by Next-Generation Sequencing in Transgenic Cattle

**DOI:** 10.1371/journal.pone.0050348

**Published:** 2012-11-21

**Authors:** Ran Zhang, Yinliang Yin, Yujun Zhang, Kexin Li, Hongxia Zhu, Qin Gong, Jianwu Wang, Xiaoxiang Hu, Ning Li

**Affiliations:** 1 State Key Laboratory of Agrobiotechnology, China Agricultural University, Beijing, China; 2 Department of Life Science, Bioyong Technologies Inc., Beijing, China; 3 Beijing GenProtein Biotech Company Ltd., Beijing, China; Huazhong Agricultural University, China

## Abstract

As the number of transgenic livestock increases, reliable detection and molecular characterization of transgene integration sites and copy number are crucial not only for interpreting the relationship between the integration site and the specific phenotype but also for commercial and economic demands. However, the ability of conventional PCR techniques to detect incomplete and multiple integration events is limited, making it technically challenging to characterize transgenes. Next-generation sequencing has enabled cost-effective, routine and widespread high-throughput genomic analysis. Here, we demonstrate the use of next-generation sequencing to extensively characterize cattle harboring a 150-kb human lactoferrin transgene that was initially analyzed by chromosome walking without success. Using this approach, the sites upstream and downstream of the target gene integration site in the host genome were identified at the single nucleotide level. The sequencing result was verified by event-specific PCR for the integration sites and FISH for the chromosomal location. Sequencing depth analysis revealed that multiple copies of the incomplete target gene and the vector backbone were present in the host genome. Upon integration, complex recombination was also observed between the target gene and the vector backbone. These findings indicate that next-generation sequencing is a reliable and accurate approach for the molecular characterization of the transgene sequence, integration sites and copy number in transgenic species.

## Introduction

The rapid development of transgenic livestock has led to new commercial opportunities in agriculture, biomedicine and environmental science. In addition, several recombinant proteins that are specifically expressed in the mammary glands of transgenic livestock, such as recombinant human antithrombin (ATryn®) and recombinant human C1 esterase inhibitor (Ruconest®), have been approved by the European Medicines Evaluation Agency (EMEA) and the United States Food and Drug Administration (FDA) and are currently on the market (http://www.gtc-bio.com/; http://www.pharming.com/). Because the production and use of transgenic livestock are likely to become more widespread, novel approaches to improve the molecular characterization of transgenes in these animals would have considerable economic and commercial benefits.

Commonly used transgenic techniques such as pronuclear injection, retroviral infection and nuclear transfer result in the random integration of multiple copies of the transgenes in the host genome [1]. The identification of integration sites is often unnecessary for a functional analysis of the transgene. Nevertheless, the random insertion of multiple copies can have marked effects, such as inactivation of an endogenous gene upon transgene insertion, different levels of transgene expression and even silencing of the transgene when inserted into a heterochromatic region which are typically greatly influenced by the chromosome position effects [2]–[5]. The potential for insertional mutagenesis of endogenous genes makes identifying the location and number of the transgenes critical for evaluating the relevance of the transgene integration site to the specific phenotype. In addition, the increasing number of transgenic livestock and, consequently, the large amount of untargeted genetic material potentially harboring transgenes highlight the need for a powerful and reliable technique to perform transgene integration site mapping to satisfy biosafety requirements.

Polymerase chain reaction (PCR)-based chromosome-walking techniques, including inverse PCR [6], ligation-mediated PCR [7], [8] and specific-primer PCR [9], [10], are the major methods that are currently used to precisely identify transgene flanking sequences. However, these techniques often produce nonspecific amplification products and are therefore incapable of reliably assessing multiple integration events [11]. Improved techniques, such as fusion primer and nested integrated PCR, have been developed to address this problem; nevertheless, only the locations of chromosomal integration sites that contain relatively few tandem copies of the transgene can be identified [12], [13]. Transgenes can often be of considerable size (e.g., >100 kb), which can make it difficult to determine whether the integrated sequence is complete. In addition, multiple copies of the transgene (or incomplete sections of the transgene) may be integrated into different genomic locations, increasing the challenge of detecting these copies.

Previously, we generated transgenic cloned cattle harboring a 150-kb bacterial artificial chromosomal (BAC) that specifically expresses human lactoferrin (hLF) in milk at a high expression level of 3.4 g/L [14]. Several studies indicate that hLF is involved in iron absorption and broad-spectrum primary defense, which suggests that hLF may have vital therapeutic applications [15], [16]. To assess the biosafety of the hLF transgene for use in commercial applications, an evaluation of the position and copy numbers of the hLF transgene is critical (http://www.fda.gov/downloads/AnimalVeterinary/GuidanceComplianceEnforcement/GuidanceforIndustry/UCM113903.pdf). Initial attempts to identify the integration site of the BAC in the bovine genome by chromosomal walking were unsuccessful (data not shown), suggesting a break in the BAC; thus, the endogenous sequences flanking the integration site and whether there was integration of multiple copies of the transgene remained unknown. Therefore, an efficient method for identifying the specific transgene integration sites was needed. Next-generation sequencing has had a profound impact on genomic research and has become a powerful tool with a diverse range of applications. Next-generation sequencing has enabled the comprehensive analysis of whole genomes in a cost-effective, routine and widespread manner [17]. In this study, we investigated the use of next-generation sequencing and subsequent bioinformatic analyses to characterize the sequence signature of the hLF BAC transgene and determine the exact insertion site(s) and copy number in three individual transgenic cattle.

**Figure 1:**
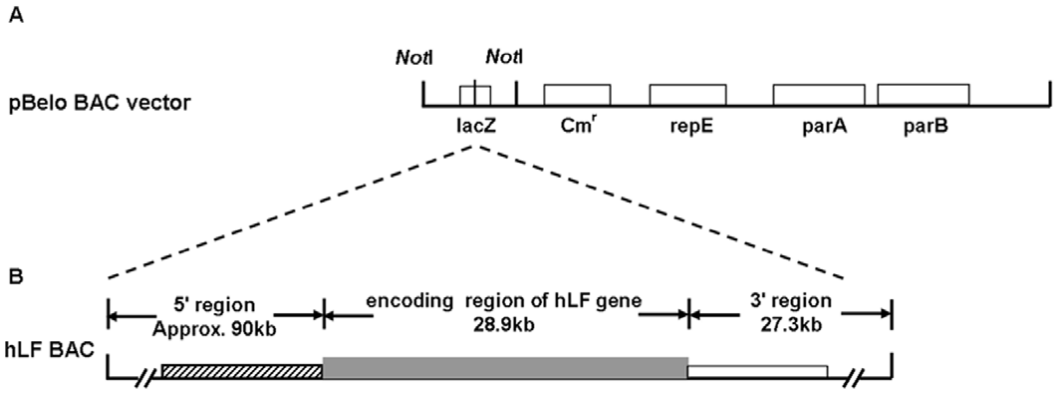
Schematic representation of the transgene constructs. (A) Structure of the transgene construct released from the pBeloBAC vector by *Not*I digestion. The transgene backbone contains the lacZ gene, the chloramphenicol resistance gene (Cm^r^) and the regulatory genes repE, parA and parB. The dotted lines indicate the insertion position of the hLF BAC into the pBeloBAC vector. (B) The hatched, gray and open boxes represent the 5′ regulatory, encoding and 3′ regulatory regions, respectively, of the hLF gene.

## Materials and Methods

### Transgenic Animals

The generation of transgenic cattle that specifically express human lactoferrin (hLF) in milk has been described previously [14]. hLF BAC clones containing the entire hLF genomic sequence (GenBank accession number: U95626) were obtained by screening a human BAC library (Genome Systems Inc.). Three transgenic cattle were detected, including the transgenic founders (F0) #040825 and #050211, which were cloned from the same fetal fibroblast cell lines, and #101026, from the second generation (F2) of #040825. Genomic DNA was extracted from ear biopsies of the three transgenic cattle with a QIAsymphony DNA Mini kit (Qiagen, German) and stored at –20°C until needed. This animal work was approved by the Institutional Animal Care and Use Committee of China Agricultural University (ID: SKLAB-2010-05-01). All surgery was performed under sodium pentobarbital anesthesia, and all efforts were made to minimize suffering.

**Figure 2:**
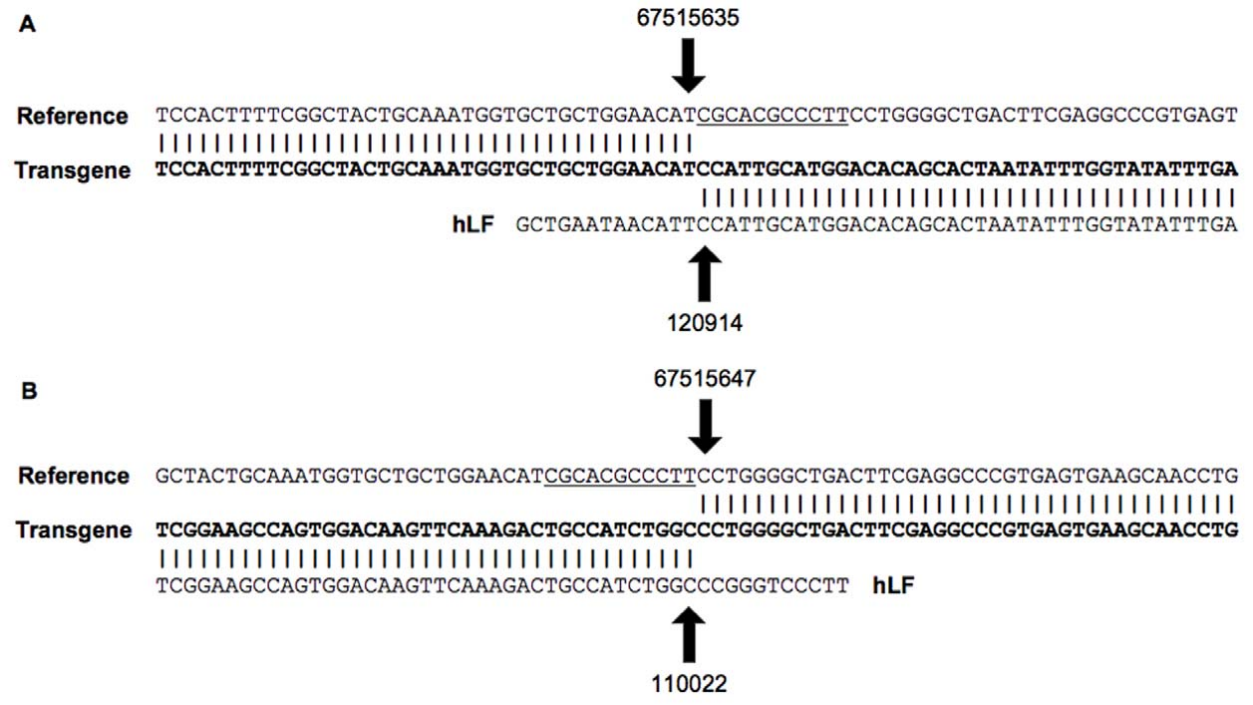
The transgene integration sites. The integration sites for the hLF BAC transgene on chromosome 15 in the bovine genome. (A) The left boundary of the integration site. (B) The right boundary of the integration site. Reference, the genomic DNA sequence of wide-type bovine; Transgene, the genomic DNA sequence of transgenic cattle; hLF, the DNA sequence of hLF BAC. The underlined regions indicate the deletion of 11 nucleotides in the genome.

### Whole Genome Sequencing

DNA was extracted from the blood of the three transgenic cattle with a QIAGEN DNA extraction kit (Qiagen Sciences, Germantown, MD). A total of 1.5 µg whole genomic DNA was sonicated with a Bioruptor sonication system (Diagenode, Inc.) to produce fragments ranging in size from 250 to 650 bp with a peak at 300–400 bp. DNA in 0.5 × TE buffer was pulsed for 21 cycles; each cycle was performed at 30 sec on and 30 sec off under high frequency. The DNA fragments were purified with a Qiagen purification kit (Qiagen Sciences, Germantown, MD). The DNA fragments were blunt end repaired and adenylated, followed by adaptor ligation according to the protocol of the Truseq DNA sample preparation kit V2 (Illumina, San Diego, CA). Size selection was performed on a 2% agarose gel. The portion of the gel corresponding to 450–550 bp DNA was excised and purified with a Qiagen gel extraction kit (Qiagen Sciences, Germantown, MD). PCR enrichment was performed for 10 cycles, followed by purification. The libraries were quantified by a LightCycler® 480‖Real-Time PCR System (Roche Diagnostics), and the insert size was measured with an Agilent 2100 (Agilent Technologies, San Diego CA). Massive parallel sequencing of the DNA libraries was applied to cBot and Hiseq2000 according to the manufacturer’s protocols (Illumina, San Diego CA). The read numbers collected for cattle #040825, #050211 and #101026 were 264 M, 246 M and 307 M, respectively.

**Figure 3:**
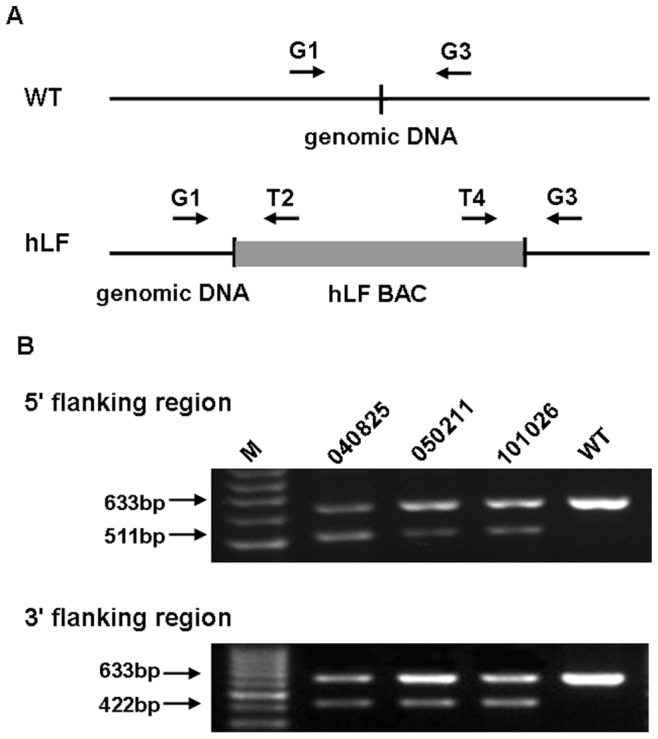
Verification of the integration sites of the transgene by PCR. (A) The arrows represent the PCR primers used for the detection of the flanking sequences. G1 and G3 are genome-specific primers, and T2 and T4 are transgene-specific primers. WT, genome of wild-type cattle; hLF, genome of transgenic cattle. (B) PCR detection of the integration sites in the three transgenic cattle and one wild-type cow. The amplified product for the wild-type sequence was 633 bp, while those for the 5′ and 3′ flanking regions of the transgenic sequence were 511 bp and 422 bp, respectively. M, 100 bp DNA ladder; WT, genome of wide-type cattle, which was used as the negative control.

### Data Analysis

Sequencing depth analysis was performed to estimate the copy numbers of the BAC and pBeloBAC vector. Briefly, low-quality reads were filtered out using custom Perl scripts with threshold Q20. All of the filtered sequencing reads were then mapped to the reference *Bos taurus* genome sequence (Bos_taurus_UMD_3.1, build 6.1), the hLF BAC (151,726 bp) and the pBeloBAC vector sequence (Genbank accession number: U51113, 7,378 bp), respectively, with Burrows-Wheeler Aligner (BWA, version 0.5.9) [18]. The unmapped reads were de novo assembled by SOAPdenovo (version 1.05) and the resultant contigs were blasted against the hLF transgene and pBeloBAC vector to find bridging reads between the host genome and the foreign fragments. To identify the interval of transgene integration, abnormal read pairs with one end mapping to the reference and the other end to the transgene or the vector were selected for further security. The exact integration breakpoints were finally identified by split-read analysis that spanning the transgene insertion junctions.

**Figure 4:**
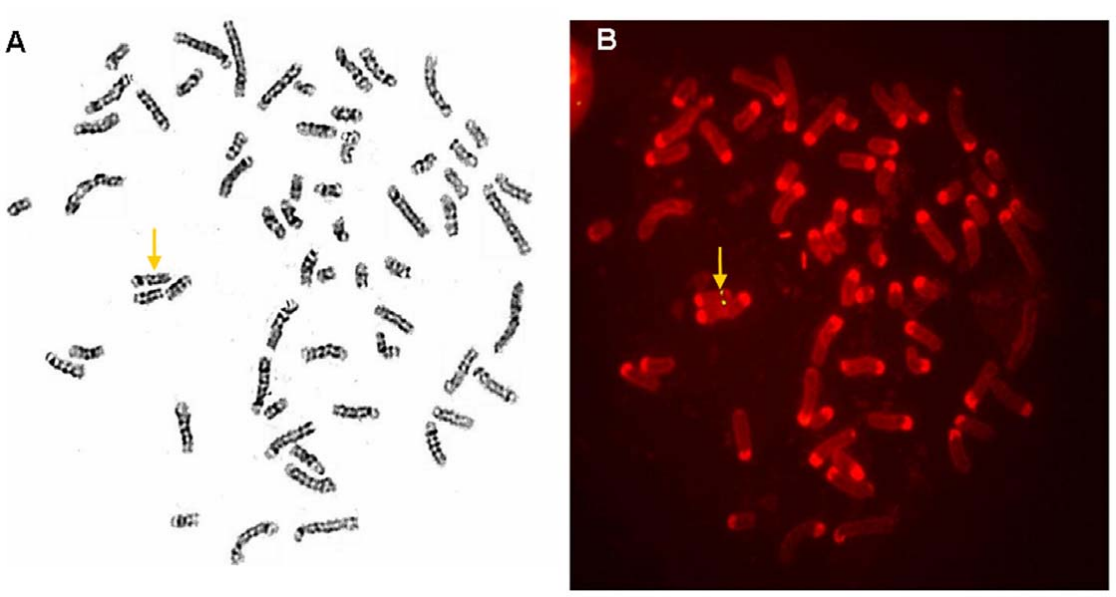
Verification of the transgene chromosomal location by FISH analysis. Detection of the transgene loci in the transgenic cow #040825 by (A) the GTG-banding pattern of metaphase spreads before hybridization and (B) the same metaphase after FISH. The arrows indicate the transgene integration site on chromosome 15.

### PCR Analysis

To verify the integration breakpoints, PCR was performed on genomic DNA samples from the three transgenic cattle and a single wild-type cow. The 5′ flanking transgene locus was identified using the primers G1 (5′-CCCAGGCAACCATTAATCAG-3′), G3 (5′-ATGCCGTTGTTGACGTTGTA-3′) and T2 (5′-CTTAGCCCATGCC TCATTGT-3′); the 3′ transgene locus was identified using the primers G1 (5′-CCCAGGCAACCATTAATCAG-3′), G3 (5′-ATGCCGTTGTTGACGTTGT A-3′) and T4 (5′-GGCTGAAAGGGACGAGTATG-3′). Primers T2 and T4 were specific to the 5′ and 3′ flanking sequences, respectively, of the transgene and could therefore detect the transgene and endogenous genomic DNA simultaneously. The products were amplified with a GeneAmp PCR System 9700 (AB, USA), with cycling parameters of 94°C for 5 minute, 30 cycles of 94°C for 30 seconds, 58°C for 30 seconds and 72°C for 1 minute, followed by a final extension at 72°C for 5 minutes.

**Figure 5:**
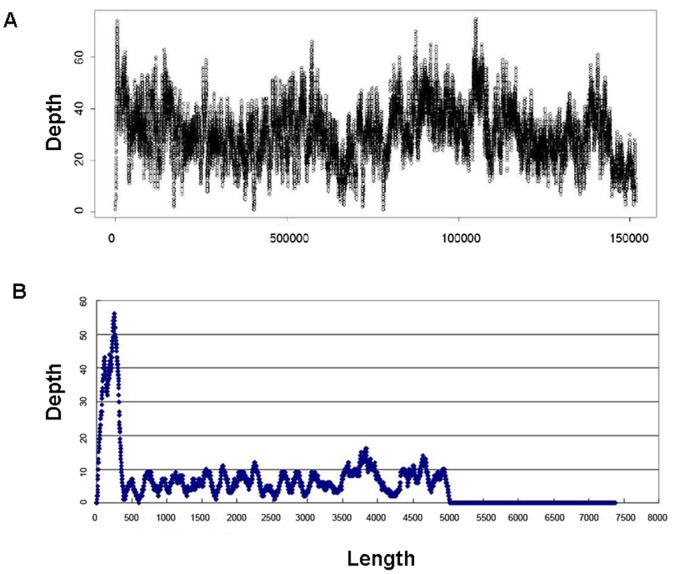
Sequencing depth of the hLF BAC and the pBeloBAC vector. By mapping the Illumina reads onto the (A) *hLF* BAC and (B) pBeloBAC vector, the sequencing depth was calculated in 10-bp sliding windows of 5 bp. The X-axis denotes the length of the (A) *hLF* BAC and (B) pBeloBAC vector in bp, and the Y-axis denotes the sequencing depth.

**Figure 6:**
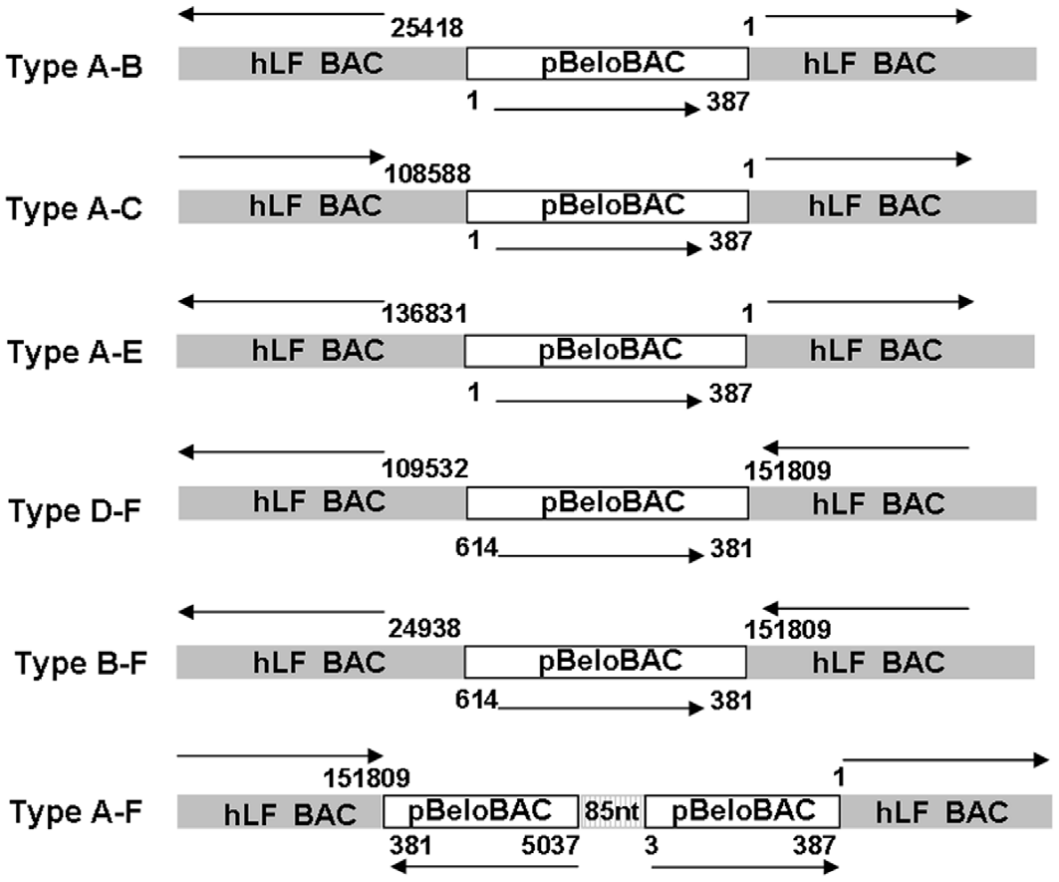
Schematic representation of the BAC rearrangements in transgenes. The positions of the junctions between the hLF BAC fragment (gray box) and the pBeloBAC vector (open box) are indicated, with arrowheads for orientation. An 85-bp unknown sequence was identified in the Type A–F molecule (hatched box). All six of these configurations should have been concatenated in an unknown format at the transgene integration site.

### GTG-binding and FISH Analysis

Ear skin fibroblasts were isolated from the three transgenic cattle as described previously [19]. The chromosomes were G-banded before hybridization using the GTG technique. Briefly, the chromosome slides were incubated at 65°C for 2 h. Trypsin treatment was performed with 0.05% trypsin in PBS for 13 seconds, and then the slides were stained with 10% Giemsa (Gibco, USA) for 8 minutes. The metaphases with the best pattern were photographed with an Olympus BX53 microscope, and the karyotype was analyzed with Karyo 3.1 software. Chromosomal integration of the transgene was demonstrated by FISH after GTG-binding. Alexa-dUTP was incorporated into a probe containing the entire linearized hLF BAC construct using the BioPrime DNA Labeling System (Invitrogen, USA), which could visualize the hybridization signal directly. The chromosome slides were counterstained with propidium iodide (Sigma, St. Louis, MO, USA) and analyzed using an Olympus BX53 microscope.

### RT-PCR Analysis

Total RNA was isolated using Trizol (Tiangen, CN) from different tissues of the transgenic (#040825) and wide-type cattle, including heart, liver, spleen, lung, kidney, abomasums, small intestine, brain and adipose. One microgram of total RNA was used for first-strand cDNA synthesis by using M-MLV Reverse Transcriptase (Promega, USA). The reaction was carried out for 1 hour at 37°C for oligodT in a total volume of 25 µl. The forward primer F (5′-AACTGCACAGCAAACCCTCT-3′) was designed spanning the exon 3 and exon 4 sequence of LDLRAD3 and the reverse primers R (5′-GTCGGCTTGGTTCAGAGACT-3′) were designed in the exon 6, giving rise to 555 bp products. A 477 bp fragment of the bovine GAPDH gene amplified by primers GAPDH F (5′-GCAAGTTCCACGGCACAG-3′) and GAPDH R (5′-CGCCAGTAGAAGCAGGGAT-3′) was used as internal control.

## Results and Discussion

### Determination of Transgene Insertion Sites by Next-Generation Sequencing

To evaluate the biosafety of the transgenic cloned cattle for commercial use, the transgene integration site(s) must be identified. The transgene in the present study is an approximately 150-kb hLF BAC ligated into the multiple cloning site of the pBeloBAC vector which was obtained by screening a human BAC library. Then the transgene construct was released from the pBeloBAC vector by *Not*I digestion and used for transfection (Figure 1). Initial attempts to identify the integration site of the BAC in the bovine genome using the widely used genome walking strategy (Clontech, USA), which employs restriction enzyme cleavage and adaptor-ligated genomic DNA fragments, were unsuccessful (data not shown). For the regular PCR-based genome walking techniques, successful amplification of the transgene depends on the restriction fragment and the random primers. In this case, the available restriction sites are unknown, resulting in nonspecific amplification or no amplification, and correspondingly, these techniques, which can be labor-intensive and prone to error, are not always reliable for characterizing transgenic animals [20]. In addition, characterizing multiple copies of transgenes throughout the host genome is also not feasible [12]. In this study, multiple and nonspecific products that could not be blasted against the bovine genome database were obtained, suggesting a break in the BAC and the integration of multiple copies of the transgene. Because the specific transgene integration sites could not be identified by PCR, we investigated the use of next-generation sequencing and subsequent bioinformatic analysis to characterize the sequence signature of the hLF BAC transgene, verify the exact insertion site(s) and determine the copy number in three individual transgenic cattle.

Initially, genomic DNA from the two founder transgenic cows was sequenced in parallel to map the hLF BAC transgene insertions. In addition, genomic DNA from cow #101026 was sequenced to evaluate the trans-generational stability of the transgene. Each DNA sample was sequenced to approximately 10× genome coverage, and the resulting data were mapped onto the bovine reference genome sequence, the hLF BAC sequence, and the pBeloBAC vector sequence, respectively. The transgene insertion sites were identified by bridging paired-end reads in which one end mapped to the bovine genome and the other end mapped to the BAC or vector regions. In all three DNA samples analyzed, a unique transgene integration site was identified on chromosome 15. Split reads spanning this region were further analyzed to map the specific integration breakpoints. The left boundary was mapped to position 67,515,635 of chromosome 15, which was flanked by position 120,914 of the hLF BAC (Figure 2A). The right boundary was located between position 67,515,647 of chromosome 15 and position 110,022 of another inserted hLF BAC (Figure 2B). All three DNA samples conformed to these specific integration breakpoints, and no alternative junction reads were identified.

### Verification of Transgene Integration Breakpoints by PCR

Once the sequence of the insertion region was identified, event-specific PCR was performed on the three DNA samples (Figure 3A). To investigate the genetic stability of the transgene, 14 other transgenic cattle, including some first generation (F1) and second generation cows, were also monitored to confirm the integration sites. Specifically, the forward primer G1 in the endogenous genome 5′ of the integration site and the reverse primer G3 in the 3′ flanking region will amplify the wild-type locus, generating 633-bp products. These primers do not amplify when the transgene is present. By contrast, G1 with the reverse primer T2 from the transgene generates a 511-bp product when the transgene is present in the 5′ flanking transgene locus, which will generate a 633-bp product from wild-type cattle and 633- and 511-bp products from the transgenic cattle. The transgenic cattle are heterozygous, with one intact chromosome from the parent and another chromosome integrated by the transgene, and the corresponding PCR products were observed as expected. Similarly, the forward primer T4 from the transgene with G3 generates a 422-bp product when the transgene is present in the 3′ flanking transgene locus, which will generate a 633-bp product from wild-type cattle and 633- and 422-bp products in the transgenic cattle. All samples analyzed by PCR exhibited the same breakpoint that had been identified by next-generation sequencing, suggesting that the transgene is stable between generations (Figure 3B and Figure S1). Furthermore, the rearrangement of genomic DNA, including deletion or translocation, has been observed at the integration sites of transgenes in previous studies [21], [22]. A deletion of an 11-nucleotide portion of the cow genome at the insertion site was also observed (67515636–67515646 of chromosome 15), which exhibited a characteristic signature of transgene integration (Figure 2).

### Verification of Transgene Chromosomal Location by FISH

GTG-banding was performed on metaphase spreads of fibroblast cells from the transgenic cattle, and more than 100 metaphase spreads were acquired from each animal. The banded metaphases were identified and photographed before hybridization, and the same metaphases were photographed again after hybridization to detect the signals. A large number of metaphase spreads must be observed because not all will display hybridization signals. In this study, approximately 50% of the metaphase spreads exhibited positive FISH signals, indicating the presence of the transgene. Next, 20 metaphase spreads that exhibited both clear GTG-banding patterns and positive FISH signals were used to confirm the identity of the signal-bearing chromosome according to the standard cattle karyotype proposed by ISCNDB 2000 [23]. As expected, positive FISH signals were observed on chromosome 15 from the DNA of #040825 (Figure 4) as well as from the DNA of #050211 and #101026 (Figures S2 and S3). These results confirm that the transgene had a single integration site on chromosome 15q26, in agreement with the next-generation sequencing results.

### Determination of Transgene Rearrangement and Copy Number

Although the sequencing coverage of the cow genome was approximately 10× for each DNA sample, the effective sequencing depth of the hLF BAC ranged from 20× to 50× (Figure 5). This discrepancy implied that multiple copies of the hLF BAC had been incorporated into the cow genome and that some copies might be incomplete. This conclusion was supported by quantitative PCR, which revealed a variable copy number in different regions of hLF BAC, from 2 to 8 (data not shown). In addition, to quantify the transgene copy number, all incidences of an abnormal paired-end read that bridged the BACs and the pBeloBAC vector were analyzed, and a complex pattern of sequencing depth distribution of the pBeloBAC vector was observed (Figure 6), suggesting that a complex rearrangement of transgenes may have occurred upon integration. Overall, six different BAC-vector junctions were identified in the transgenic cattle (Figure S4). The inserted vector sequences were much shorter than the BAC inserts, and hence long-range inverse PCR primers were used to elucidate the arrangement of these BACs. Sequencing of the specific PCR products revealed that six of these configurations should have been concatenated in an unknown format in the transgenic cattle genome (Figure 6), suggesting that these BACs had been rearranged during or subsequent to transgene integration. We assume that this rearrangement is the critical barrier to determining the integration sites by the PCR-based techniques. It has been shown previously that transgene concatemers tend to exist as head-to-tail arrays, which is consistent with the order of repetitive DNA in the host genome [24]. Our results indicate that the formation of transgene concatemers may not always be similar to the order of repetitive DNA in animal genomes.

### Expression of the Endogenous Gene in the Transgenic Cattle

The transgene was integrated into the intron 4 of low density lipoprotein receptor class A domain containing 3 (LDLRAD3) gene according to the exact position, which contains six coding exons and five introns. This gene is located in the left boundary of a 6.6Mb gene desert region to the 3′ direction, where no protein-coding genes existed. LDLRAD3 plays a central role in mammalian cholesterol metabolism and the receptor protein binds LDL and transports it into cells by endocytosis [25]. To evaluate whether the transgene affect the expression of the LDLRAD3 gene, the endogenous LDLRAD3 mRNA expression in different tissues of transgenic cattle #040825 was analyzed by RT-PCR (Figure S5). LDLRAD3 transcripts yielded an expected 555 bp size band and the transcriptional profiling of transgenic cattle is similar to that of wide-type cattle. This result confirmed that the integration of hLF BAC did not affect the expression of endogenous gene.

### Conclusion

To date, PCR-based techniques have been widely used for precise transgene flanking sequence identification in biological research, but these techniques are limited in their ability to identify the specific amplification of a transgene that is present in multiple copies or as an incomplete sequence. The present study has demonstrated the successful use of a high-throughput next-generation sequencing platform to characterize transgene integration. This approach identified both complete and incomplete hLF BAC integration sites with high specificity at single nucleotide resolution and also provided information on the chromosomal location and transgene copy number. Each application of this next-generation sequencing approach was verified by commonly used techniques for transgene characterization-PCR for the integration sites and FISH for the chromosomal location–and shown to be accurate and consistent. In addition, high-throughput sequencing enabled the determination of the copy number of both the integrated transgene and the backbone of the vector by counting the relative sequencing depths of the corresponding DNA regions. Furthermore, when combined with PCR at specific locations, this approach clarified whether the transgene had integrated into the genome as a complete copy or as an incomplete fragment. The future application of high-throughput sequencing to the characterization of transgenic animals and plants will be of profound significance and is likely to complement, if not replace, traditional PCR-based methods.

## Supporting Information

Figure S1 **Verification of the integration sites of the transgene by PCR.** PCR detection of the (A) 5′ flanking region and (B) 3′ flanking region of the hLF BAC transgene in fourteen transgenic cattle and one wild-type cow. The amplified product for the wild-type sequence was 633 bp, while those for the 5′ and 3′ flanking regions of the transgenic sequence were 511 bp and 422 bp, respectively. M, 100 bp DNA ladder; WT, genome of wild-type cattle.
(TIF)

Figure S2 **Verification of the transgene chromosomal location by FISH analysis.** Detection of the transgene loci in transgenic cow #050211 by (A) the GTG-banding pattern of metaphase spreads before hybridization and (B) the same metaphase after FISH. The arrows indicate the transgene integration site on chromosome 15.
(TIF)

Figure S3 **Verification of the transgene chromosomal location by FISH analysis.** Detection of the transgene loci in transgenic cow #101026 by (A) the GTG-banding pattern of metaphase spreads before hybridization and (B) the same metaphase after FISH. The arrows indicate the transgene integration site on chromosome 15.
(TIF)

Figure S4 **Schematic representation of the BAC-vector junction structures.** Within the transgene integration site, six different BAC-vector junction structures were identified by analyzing the bridging read-pair data. The positions of the junctions between the hLF BAC fragment (gray box) and the pBeloBAC vector (open box) are indicated, with arrowheads for orientation.
(TIF)

Figure S5 **RT-PCR analysis of LDLRAD3 expression.** RT-PCR was performed to detect the LDLRAD3 mRNA expression in different tissues of the transgenic and wild-type cattle. The transcripts for the LDLRAD3 and GAPDH were 555 bp and 477 bp, respectively. M, 250-bp DNA ladder; hLF, transgenic cattle of #040825; WT, wide-type cattle. Bovine GAPDH gene was used as internal control.
(TIF)
