# Supplementary figures and images for: Molecular Characterization of Transgene Integration by Next-Generation Sequencing in Transgenic Cattle
Source: PLoS One. 2012 Nov 21;7(11):e50348. doi: 10.1371/journal.pone.0050348 (PMC3503979; doi:10.1371/journal.pone.0050348)

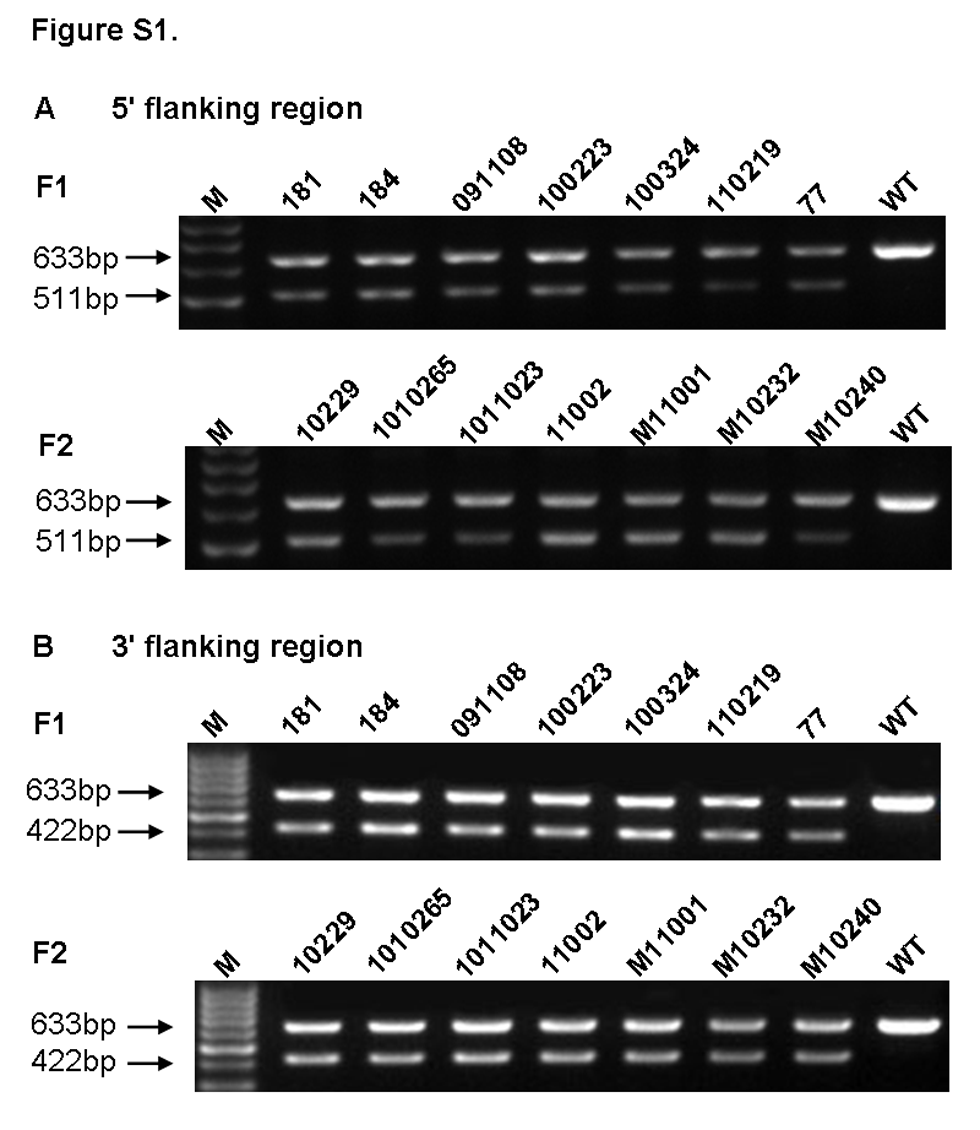

Supplement: Figure S1 — Verification of the integration sites of the transgene by PCR. PCR detection of the (A) 5′ flanking region and (B) 3′ flanking region of the hLF BAC transgene in fourteen transgenic cattle and one wild-type cow. The amplified product for the wild-type sequence was 633 bp, while those for the 5′ and 3′ flanking regions of the transgenic sequence were 511 bp and 422 bp, respectively. M, 100 bp DNA ladder; WT, genome of wild-type cattle. (TIF) [file pone.0050348.s001.tif]

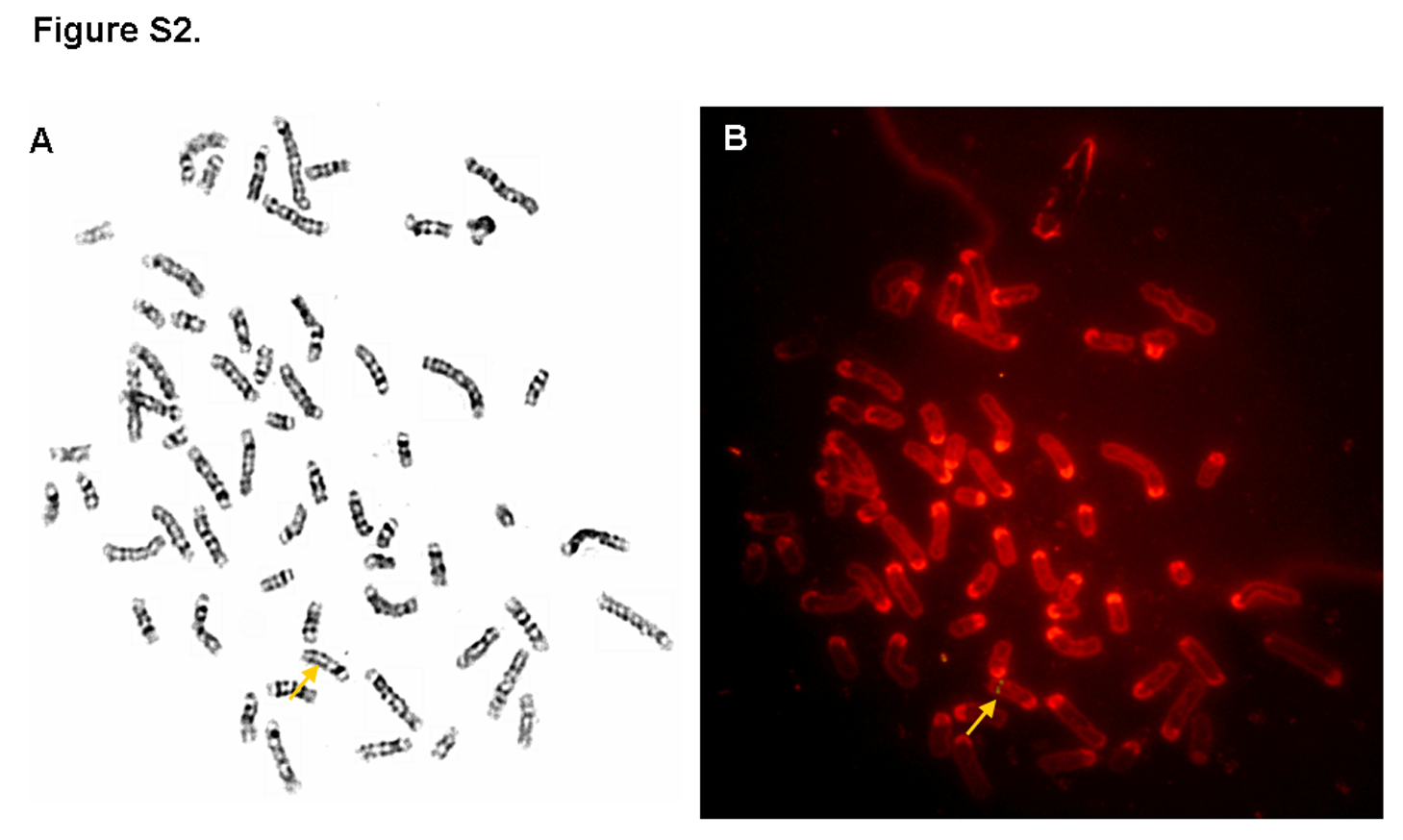

Supplement: Figure S2 — Verification of the transgene chromosomal location by FISH analysis. Detection of the transgene loci in transgenic cow #050211 by (A) the GTG-banding pattern of metaphase spreads before hybridization and (B) the same metaphase after FISH. The arrows indicate the transgene integration site on chromosome 15. (TIF) [file pone.0050348.s002.tif]

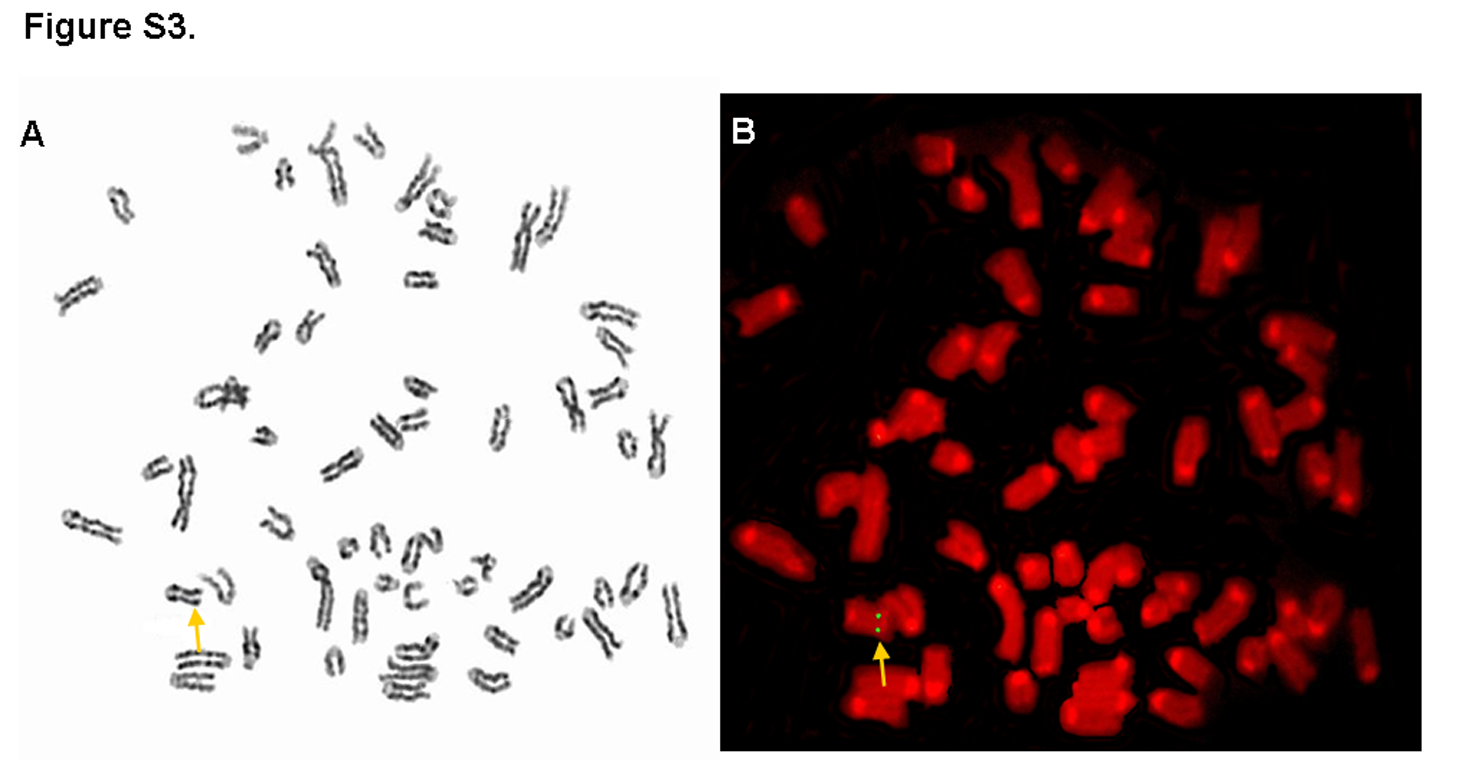

Supplement: Figure S3 — Verification of the transgene chromosomal location by FISH analysis. Detection of the transgene loci in transgenic cow #101026 by (A) the GTG-banding pattern of metaphase spreads before hybridization and (B) the same metaphase after FISH. The arrows indicate the transgene integration site on chromosome 15. (TIF) [file pone.0050348.s003.tif]

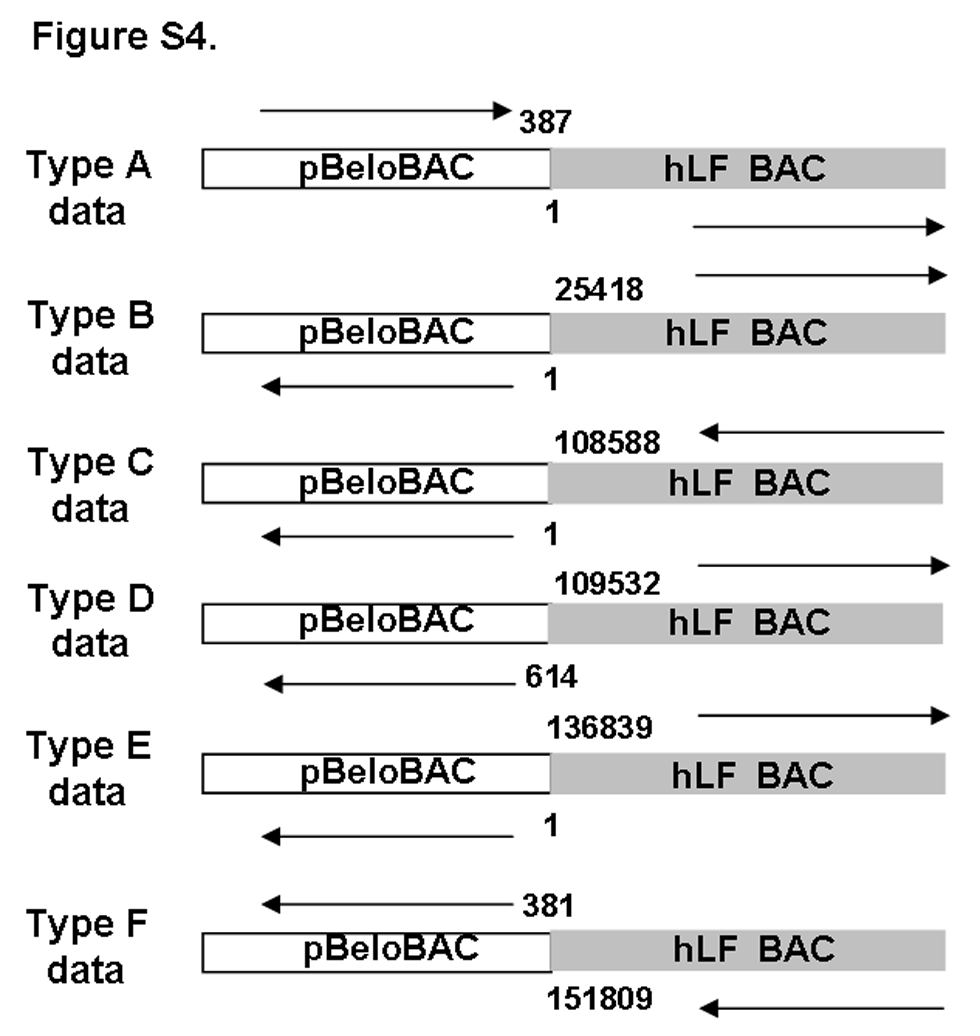

Supplement: Figure S4 — Schematic representation of the BAC-vector junction structures. Within the transgene integration site, six different BAC-vector junction structures were identified by analyzing the bridging read-pair data. The positions of the junctions between the hLF BAC fragment (gray box) and the pBeloBAC vector (open box) are indicated, with arrowheads for orientation. (TIF) [file pone.0050348.s004.tif]

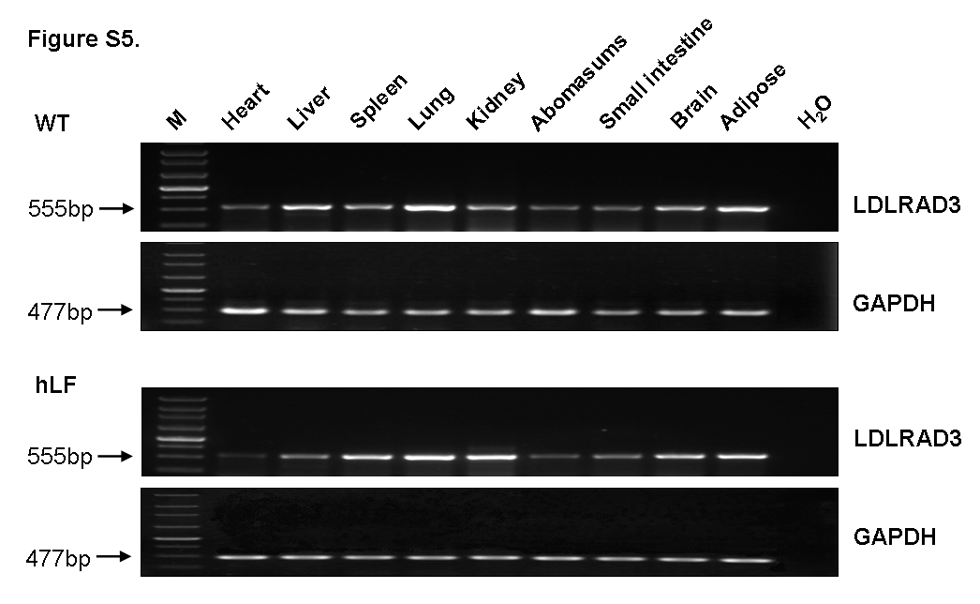

Supplement: Figure S5 — RT-PCR analysis of LDLRAD3 expression. RT-PCR was performed to detect the LDLRAD3 mRNA expression in different tissues of the transgenic and wild-type cattle. The transcripts for the LDLRAD3 and GAPDH were 555 bp and 477 bp, respectively. M, 250-bp DNA ladder; hLF, transgenic cattle of #040825; WT, wide-type cattle. Bovine GAPDH gene was used as internal control. (TIF) [file pone.0050348.s005.tif]
